# Is There a Correlation Between Patient-Reported Bath Ankylosing Spondylitis Disease Activity Index (BASDAI) Score and MRI Findings in Axial Spondyloarthropathy in Routine Clinical Practice?

**DOI:** 10.7759/cureus.19626

**Published:** 2021-11-16

**Authors:** Swetha Byravan, Nibha Jain, Jenna Stairs, Winston Rennie, Arumugam Moorthy

**Affiliations:** 1 Rheuamtology, University Hospitals of Leicester National Health Service Trust, Leicester, GBR; 2 Rheumatology, University Hospitals of Leicester National Health Service Trust, Leicester, GBR; 3 Radiology, University Hospitals of Leicester National Health Service Trust, Leicester, GBR; 4 College of Life Sciences, University of Leicester, Leicester, GBR

**Keywords:** sacroilitis, disease activity, patient reported outcomes, basdai, mri, axial spondyloarthropathy

## Abstract

Background

The Bath Ankylosing Spondylitis Disease Activity Index (BASDAI) is the patient-reported outcome (PRO) that is routinely used in clinical practice to monitor and measure disease activity in axial spondyloarthropathy (axSpA). BASDAI scores greater than four are thought to indicate active disease and require better control. Magnetic resonance imaging (MRI) is the most objective measure of disease activity in axSpA with its ability to pick up active inflammation both in the spine and sacroiliac joints. Previous studies have shown conflicting correlations between BASDAI and MRI, and therefore, there is the question of whether BASDAI is the best tool to monitor disease activity when it is subjective and potentially influenced by other patient factors. We, therefore, conducted a retrospective study to investigate the correlation between BASDAI and MRI in axSpA patients.

Methodology

Data were collected by retrospective analysis of axSpA patients attending University of Leicester (UHL) axSpA services. BASDAI scores were done within a year and closest to the time of MRI spine + sacroiliac joints were collected. The results prior to the initiation of biologic therapy were used. Data of one hundred and forty-nine patients were collected on their MRI results and BASDAI scores. Data were analysed using Statistical Package for the Social Sciences (SPSS) software and Pearson’s chi-squared applied to assess the correlation between BASDAI and MRI findings.

Results

Out of one hundred and forty-nine patients, 61.7% had active sacroiliitis on their MRI, 57.7% had chronic sacroiliitis, 53% had active spinal inflammation, and 17.4% had other MRI findings of active disease. There was a significant correlation between active sacroiliitis and BASDAI (p=0.014), but similar results were not found with other radiological features. A significant correlation was also found with males having higher BASDAI scores compared to females (p=0.027).

Conclusion

This study demonstrates a statistically significant correlation between BASDAI and active sacroiliitis with those having higher scores more likely to have active disease on their MRI.

## Introduction

Axial spondyloarthritis (axSpA) is an umbrella term that entails a chronic rheumatological inflammatory condition, predominantly affecting the axial skeleton. It encompasses both radiological axSpA also known as ankylosing spondylitis (AS), where evidence of disease is present on x-ray, and non-radiological where it is not. In non-radiological axSpA disease, patients have clinical features consistent with axSpA and are human leukocyte antigen (HLA)-B27 positive and/or have suggestive inflammation evidenced on magnetic resonance imaging (MRI) thus fulfilling the Assessment of SpondyloArthritis International Society (ASAS) criteria [1].

Management of axSpA involves the use of medication such as non-steroidal anti-inflammatory drugs (NSAIDs) and biologic therapy. Current British Society of Rheumatology guidelines advise monitoring disease by using Bath Ankylosing Spondylitis Disease Activity Index (BASDAI), which is a patient-reported outcome (PRO). BASDAI involves the patient to self-score on various features including fatigue, stiffness, and pain [2]. This is then calculated into a score and a score of four or more indicates active disease. In routine clinical practice, BASDAI is largely subjective and based on the patient’s perspective of their disease. Though it is important to acknowledge and incorporate patient views into shared decision making, the patient-reported disease activity scores are inevitably influenced by other confounding factors such as age, disease duration, mood, comorbid conditions, and chronic pain [3-5]. As a clinician, it is important to consider whether a high BASDAI score is a true reflection of disease as it has a significant impact on subsequent treatment decisions.

MRI is the most objective tool currently available to measure disease activity with its ability to identify active inflammation both in the sacroiliac joints (SIJs) and axial spine in axSpA. As MRI becomes more accessible and available, one must consider how this tool can be used in treatment decisions and whether it correlates to subjective BASDAI scores. Currently, the European Alliance of Associations for Rheumatology (EULAR) recommends it as not only the imaging modality of choice for diagnosis but also disease monitoring [6]. There are few studies that have investigated this relationship, which has found either no or weak correlation between BASDAI and MRI [7,8]. Furthermore, MRI used in clinical trials to assess the long-term radiological response of biologic therapy have found significant radiological improvement on MRI that correlated poorly with BASDAI scores [9,10].

The unresolved question of correlation between BASDAI and MRI makes one re-assess the accuracy of BASDAI and whether this is really the best tool to monitor and make treatment decisions in axSpA. To further investigate this, we conducted a single-centre cross-sectional study on patients with a known diagnosis on axSpA and compared their BASDAI score with MRI findings. We also looked at whether there was a statistical correlation between other demographic and extra-articular clinical features with the BASDAI value.

## Materials and methods

Data were collected by retrospective analysis of axSpA patients attending University of Leicester (UHL) axSpA services. This was obtained from the UHL axSpA database of patients with known diseases.

Inclusion criteria were patients with a diagnosis of axSpA with a documented BASDAI within a year of MRI spine + SIJ, prior to starting any biological treatment. Exclusion criteria were anyone with active infection and/or malignancy, BASDAI not documented prior to starting biological therapy or within a year of MRI, or any patient where it was not possible to obtain all information regarding clinical features or HLA-B27 status. Patients with secondary fibromyalgia were not specifically excluded. 

For all patients’ demographic characteristics were collected such as age, gender, and ethnicity from the online trust-based electronic systems. Electronic clinic letters were used to collate data regarding clinical features including seronegative features (uveitis, inflammatory back pain, enthesitis, peripheral arthritis, dactylitis, psoriasis, and inflammatory bowel disease), family history, and response to NSAIDs. Electronic pathology results were used to identify baseline C-reactive peptide (CRP), which was defined to be CRP at the time of diagnosis and HLA-B27 status.

MRI was performed as per axial spondyloarthropathy protocol, and the data were viewed and analysed using online radiological reports stored on picture archiving and communication system (PACS) software. This was used to identify active sacroiliitis, chronic sacroiliitis, active axial disease, and other radiographic changes of significance, such as costovertebral, costotransverse, and sternoclavicular inflammation. Active disease was defined to be the presence of active inflammation in the form of bone marrow oedema either in the SIJ and/or spine.

Data of one hundred and forty-nine patients were collected all whom met the inclusion criteria.

The BASDAI score of all patients was then compared to their corresponding MRI findings. BASDAI was also compared with demographic data, clinical features, and laboratory results. Data were analysed using Statistical Package for the Social Sciences (SPSS) software and Pearson’s chi-squared test applied to calculate p-values between BASDAI and corresponding datasets.

## Results

Out of 149 patients, 65.7% (98) were male and 34.3% (51) female. The mean age of the patient was 43 years. There was a statistically significant correlation between gender and BASDAI (p=0.027) with males having a greater BASDAI score >4 compared to females. Summary of demographic data with corresponding p values in relation to correlation with BASDAI is summarised in Table 1.

Out of 149, 61.7% (92) had active sacroiliitis on their MRI, 57.7% (86) had chronic sacroiliitis, and 53% (79) had active spinal inflammation. 17.4% (26) had other MRI findings of active disease including costovertebral and transverse inflammation, axial enthesitis, and sternoclavicular inflammation. There was a significant correlation between active sacroiliitis and BASDAI (p=0.014), but similar results were not found with other radiological features; chronic sacroiliitis (p=0.85), active axial disease (p=0.107), or other diseases (p=0.060). Radiological results are summarised in Table 2.

**Table 1:** Summary of demographic features and statistical correlation with BASDAI. BASDAI: Bath Ankylosing Spondylitis Disease Activity Index.

	Number of patients	Correlation to BASDAI
Ethnicity		p=0.668
Caucasian	102	
Asian	42	
Afro-Caribbean	2	
Gender		p=0.027
Female	51	
Male	98	

**Table 2:** Radiographic results and correlation with BASDAI. BASDAI: Bath Ankylosing Spondylitis Disease Activity Index.

	Number of patients	Correlation with BASDAI
Active sacroiliitis	92	p=0.014
Chronic sacroiliitis	86	p=0.85
Active axial disease	79	p=0.107
Other active disease	26	p=0.704

## Discussion

BASDAI and MRI

BASDAI is a subjective score of disease activity recorded by patients on their interpretation of disease severity compiled of multiple factors. A score of four or more is thought to be significant and one that requires better disease control. However, the question arises of whether this subjective assessment correlates accurately to objective inflammation on MRI or is the score skewed by other patient factors and therefore can it accurately assess disease activity.

In this study, we compared the BASDAI scores that were most closely recorded to the timing of MRI and within a year of the scan. They were all taken prior to initiation of any biological therapy, usually close to the time of diagnosis to reduce the risk of confounding factors influencing the data. The major finding from this study is that it did demonstrate a statistically significant relationship between active sacroiliitis and BASDAI. This however did not correlate to other radiographic findings of the active axial disease, chronic sacroiliitis, or other diseases, which is the first time that this relationship has been studied. Nevertheless, the study suggests that a BASDAI score of more than four does correlate well to active sacroiliac disease, which provides a new welcome and encouraging data to quite a limited area of research.

A limitation of our study is that we only looked at if active sacroiliitis was present or not, we did not look at whether the extent of inflammation also correlates with BASDAI. Radiographic sacroiliitis can be quantified using the Spondyloarthritis Research Consortium of Canada (SPARCC) to provide a radiological disease activity score. Mackay et al. looked at whether there was a relationship with BASDAI [7]. They found no correlation between SPARCC and BASDAI but did find a more significant correlation with the Ankylosing Spondylitis Disease Activity Score (ASDAS)-CRP, which uses similar parameters as BASDAI but incorporates the CRP value in the calculation making it more objective. However, the sample size was small only involving 40 patients.

Zhang et al. more closely investigated the correlation between clinical and MRI indices of sacroiliitis activity using apparent diffusion coefficient (ADC) as well as the Spondyloarthritis Research Consortium of Canada (SPARCC) score in a AS group compared to a control group [11]. They found the BASDAI score showed a statistically significant correlation with ADC and SPARCC. In addition, a different study by Zhang et al. used dynamic contrast-enhanced MRI (DCE-MRI) to differentiate active and inactive stages of sacroiliitis in AS and found a significant correlation with BASDAI with those with the more active disease having a greater score [12]. Akdeniz also evaluated BASDAI as a tool for disease activity and compared it to MRI [13]. They found the positive predictive value of BASDAI to be 100% but the negative predictive value to be much lower at 15% but this was higher than other considered disease activity tools including quantitative scintigraphy, CRP, and erythrocyte sedimentation rate (ESR).

In our study, BASDAI is only compared with MRI findings at one point in time. We did not compare it longitudinally or after initiation of biologic therapy, this is due to the nature of assessment carried out in routine clinical practice due to logistic reasons. Nevertheless, it does not ascertain whether BASDAI continues to correlate with MRI findings. The Devenir des Spondylarthropathies Indifferénciées Récentes (DESIR) study conducted a two-year longitudinal study to investigate this and found there was only a statistically significant relationship in males between ASDAS and MRI SIJ, there was no relationship with BASDAI [14]. Goh et al. looked at patients with a longer disease duration of greater than 10 years and found they had on average very little activity on their MRI, but the average BASDAI score was 4.40, which would indicate active disease [8]. There was a poor correlation between the two and therefore, they suggest utilising MRI along with other measures of disease activity in the monitoring of long-term symptomatic axSpA patients.

In terms of biologic therapy, Weiß et al. evaluated the correlation between objective and subjective measures of inflammation in axSpA treated with biologic therapy-etanercept (ETA) or adalimumab (ADA) [15]. They found patients with <4 years of disease demonstrated a significantly better BASDAI improvement, and BASDAI correlated strongly with improvement in MRI SIJ scores (p=0.01) than those with longer disease duration. This suggests that symptoms may be mainly driven by inflammation in the early course of the disease but as the duration increases other factors such as secondary fibromyalgia, chronic muscle imbalance, non-physiological stress, or impact on joints can also affect patient-reported outcomes (PROs).

We did not include the relationship between ASDAS and MRI to assess if it is superior to BASDAI given the incorporation of inflammatory marker (ESR/CRP) into its score. ASDAS might be a more sensitive tool as reported by several studies previously especially monitoring response to tumour necrosis factor (TNF) inhibitors. Both Pedersen et al. and Braun et al. found ASDAS to demonstrate high responsiveness in their patient population treated with TNF inhibitors who were followed for one to two years compared to BASDAI [16,9]. Similarly, Machado et al. found ASDAS to correlate with MRI findings in AS patients treated with TNF inhibitors but also found CRP improvements to be strongly correlated with radiographic improvements [17]. We did not investigate the correlation with CRP in our study. However, we took the result at baseline rather than the closest reading to the recorded BASDAI, and we did not look at it longitudinally to see if it does improve with treatment. In our cohort, we do not check CRP at regular intervals like in other centres.

BASDAI and demographics

Results did show a significant relationship between BASDAI and gender with males scoring higher than females, which contrasts with previously published studies. A recent study by Maguire et al. found that females had a worse BASDAI score, and they scored themselves significantly worse than males across all components with the highest mean score for fatigue while males scored highest for spinal pain [18]. This is in keeping with another study by Nas et al., which included almost 80 more female patients than males but again found PRO were worse in females across multiple categories and patient-reported quality of life was better in males [19]. A review article by Rusman et al. investigated potential reasons why women score higher and found longer delays in diagnosis, lower treatment efficacy to TNF inhibitors, and lower drug adherence to be significant causes [20]. The finding in our study could be attributed to there being almost twice as many male patients as females and therefore, influencing the results. However, in our study, the BASDAI scores were taken prior to initiation of any biological therapy and usually at diagnosis. Therefore, it could be argued that male patients have a greater disease burden prior to initiation of therapy and the BASDAI reflects this as it is demonstrated in the literature that males have a greater radiographic disease burden at diagnosis and progression and BASDAI in this study correlates with active sacroiliitis [21,22].

## Conclusions

This study demonstrated a statistically significant relationship between active sacroiliitis and BASDAI indicating it is a valid measure of disease activity. However, its strength in long-term disease or after initiation of biological therapy cannot be verified in this study. ASDAS may be a better measure of disease due to its incorporation of an inflammatory marker. However, larger studies are needed with a more heterogeneous population group to ascertain which is superior and to obtain further meaningful results regarding demographics and clinical features with disease activity tools.

## References

[1] Sieper J, Rudwaleit M, Baraliakos X (2009). The assessment of spondyloarthritis international society (ASAS) handbook: a guide to assess spondyloarthritis. Ann Rheum Dis.

[2] Hamilton L, Barkham N, Bhalla A (2017). BSR and BHPR guideline for the treatment of axial spondyloarthritis (including ankylosing spondylitis) with biologics. Rheumatology (Oxford).

[3] Rudwaleit M, Listing J, Brandt J, Braun J, Sieper J (2004). Prediction of a major clinical response (BASDAI 50) to tumour necrosis factor alpha blockers in ankylosing spondylitis. Ann Rheum Dis.

[4] Zhao SS, Jones GT, Macfarlane GJ, Hughes DM, Moots RJ, Goodson NJ (2021). Association between comorbidities and disease activity in axial spondyloarthritis: results from the BSRBR-AS. Rheumatology (Oxford).

[5] Macfarlane GJ, MacDonald RI, Pathan E (2018). Influence of co-morbid fibromyalgia on disease activity measures and response to tumour necrosis factor inhibitors in axial spondyloarthritis: results from a UK national register. Rheumatology (Oxford).

[6] Mandl P, Navarro-Compán V, Terslev L (2015). EULAR recommendations for the use of imaging in the diagnosis and management of spondyloarthritis in clinical practice. Ann Rheum Dis.

[7] MacKay JW, Aboelmagd S, Gaffney JK (2015). Correlation between clinical and MRI disease activity scores in axial spondyloarthritis. Clin Rheumatol.

[8] Goh L, Suresh P, Gafoor A, Hughes P, Hickling P (2008). Disease activity in longstanding ankylosing spondylitis: a correlation of clinical and magnetic resonance imaging findings. Clin Rheumatol.

[9] Braun J, Baraliakos X, Hermann KG (2012). Golimumab reduces spinal inflammation in ankylosing spondylitis: MRI results of the randomised, placebo-controlled GO-RAISE study. Ann Rheum Dis.

[10] Sieper J, Baraliakos X, Listing J, Brandt J, Haibel H, Rudwaleit M, Braun J (2005). Persistent reduction of spinal inflammation as assessed by magnetic resonance imaging in patients with ankylosing spondylitis after 2 yrs of treatment with the anti-tumour necrosis factor agent infliximab. Rheumatology (Oxford).

[11] Zhang P, Yu K, Guo R, Shah S, Morelli JN, Runge VA, Li X (2015). Ankylosing spondylitis: correlations between clinical and MRI indices of sacroiliitis activity. Clin Radiol.

[12] Zhang M, Zhou L, Huang N, Zeng H, Liu S, Liu L (2017). Assessment of active and inactive sacroiliitis in patients with ankylosing spondylitis using quantitative dynamic contrast-enhanced MRI. J Magn Reson Imaging.

[13] Akdeniz O, Alayli G, Tosun FC (2008). Early spondyloarthropathy: scintigraphic, biological, and clinical findings in MRI-positive patients. Clin Rheumatol.

[14] Navarro-Compán V, Ramiro S, Landewé R, Dougados M, Miceli-Richard C, Richette P, van der Heijde D (2016). Disease activity is longitudinally related to sacroiliac inflammation on MRI in male patients with axial spondyloarthritis: 2-years of the DESIR cohort. Ann Rheum Dis.

[15] Weiß A, Song IH, Haibel H, Listing J, Sieper J (2014). Good correlation between changes in objective and subjective signs of inflammation in patients with short-but not long duration of axial spondyloarthritis treated with tumor necrosis factor-blockers. Arthritis Res Ther.

[16] Pedersen SJ, Sørensen IJ, Hermann KG (2010). Responsiveness of the Ankylosing Spondylitis Disease Activity Score (ASDAS) and clinical and MRI measures of disease activity in a 1-year follow-up study of patients with axial spondyloarthritis treated with tumour necrosis factor alpha inhibitors. Ann Rheum Dis.

[17] Machado P, Landewé RB, Braun J (2012). MRI inflammation and its relation with measures of clinical disease activity and different treatment responses in patients with ankylosing spondylitis treated with a tumour necrosis factor inhibitor. Ann Rheum Dis.

[18] Maguire S, Gallagher P, O'Shea F (2021). O11 Examining BASDAI by gender: differing scores, but similar patterns of disease. Rheumatology (Oxford).

[19] Nas K, Kiliç E, Tekeoğlu İ (2021). The effect of gender on disease activity and clinical characteristics in patients with axial psoriatic arthritis. Mod Rheumatol.

[20] Rusman T, van Vollenhoven RF, van der Horst-Bruinsma IE (2018). Gender differences in axial spondyloarthritis: women are not so lucky. Curr Rheumatol Rep.

[21] Ortolan A, van Lunteren M, Ramiro S (2018). Are gender-specific approaches needed in diagnosing early axial spondyloarthritis? Data from the spondyloarthritis caught early cohort. Arthritis Res Ther.

[22] Wright GC, Kaine J, Deodhar A (2020). Understanding differences between men and women with axial spondyloarthritis. Semin Arthritis Rheum.

